# Gauging the Purported Costs of Public Data Archiving for Long-Term Population Studies

**DOI:** 10.1371/journal.pbio.1002432

**Published:** 2016-04-08

**Authors:** Simon Robin Evans

**Affiliations:** Department of Evolutionary Biology and Environmental Studies, University of Zurich, Zurich, Switzerland

## Abstract

It was recently proposed that long-term population studies be exempted from the expectation that authors publicly archive the primary data underlying published articles. Such studies are valuable to many areas of ecological and evolutionary biological research, and multiple risks to their viability were anticipated as a result of public data archiving (PDA), ultimately all stemming from independent reuse of archived data. However, empirical assessment was missing, making it difficult to determine whether such fears are realistic. I addressed this by surveying data packages from long-term population studies archived in the Dryad Digital Repository. I found no evidence that PDA results in reuse of data by independent parties, suggesting the purported costs of PDA for long-term population studies have been overstated.

## Abstract 2

Ecologists were recently advised that primary data archiving threatens the sustainability of long-term population studies because of negative impacts of data reuse by others. However, this Perspective describes an empirical assessment that finds little evidence of data reuse, suggesting that such fears may be unfounded.

Data are the foundation of the scientific method, yet individual scientists are evaluated via novel analyses of data, generating a potential conflict of interest between a research field and its individual participants that is manifested in the debate over access to the primary data underpinning published studies [1–5]. This is a chronic issue but has become more acute with the growing expectation that researchers publish the primary data underlying research reports (i.e., public data archiving [PDA]). Studies show that articles publishing their primary data are more reliable and accrue more citations [6,7], but a recent opinion piece by Mills et al. [2] highlighted the particular concerns felt by some principal investigators (PIs) of long-term population studies regarding PDA, arguing that unique aspects of such studies render them unsuitable for PDA. The "potential costs to science" identified by Mills et al. [2] as arising from PDA are as follows:

- Publication of flawed research resulting from a "lack of understanding" by independent researchers conducting analyses of archived data
- Time demands placed on the PIs of long-term population studies arising from the need to correct such errors via, e.g., published rebuttals
- Reduced opportunities for researchers to obtain the skills needed for field-based data collection because equivalent long-term population studies will be rendered redundant
- Reduced number of collaborations
- Inefficiencies resulting from repeated assessment of a hypothesis using a single dataset

Each "potential cost" is ultimately predicated on the supposition that reuse of archived long-term population data is common, yet the extent to which this is true was not evaluated. To assess the prevalence of independent reuse of archived data—and thereby examine whether the negative consequences of PDA presented by Mills et al. [2] may be realised—I surveyed datasets from long-term population studies archived in the Dryad Digital Repository (hereafter, Dryad). Dryad is an online service that hosts data from a broad range of scientific disciplines, but its content is dominated by submissions associated with ecological and evolutionary biological research [8]. I examined all the Dryad packages associated with studies from four journals featuring ecological or evolutionary research: *The American Naturalist*, *Evolution*, *Journal of Evolutionary Biology*, and *Proceedings of the Royal Society B*: *Biological Sciences* (the latter referred to hereafter as *Proceedings B*). These four journals together represent 23.3% of Dryad's contributed packages (as of early February 2016). Mills et al. [2] refer to short- versus long-term studies but do not provide a definition of this dichotomy. However, the shortest study represented by their survey lasted for 5 years, so I used this as the minimum time span for inclusion in my survey. This cut-off seems reasonable, as it will generally exclude studies resulting from single projects, such that included datasets likely relate to studies resulting from a sustained commitment on the part of researchers—although one included package contains data gathered via “citizen science” [9], and two others contain data derived from archived human population records [10,11]. However, as these datasets cover extended time spans and were used to address ecological questions [12–14], they were retained in my survey sample. Following Mills et al. [2], my focus was on population studies conducted in natural (or seminatural) settings, so captive populations were excluded. Because I was assessing the reuse of archived data, I excluded packages published by Dryad after 2013: authors can typically opt to impose a 1-year embargo, and articles based on archived data will themselves take some time to be written and published.

Of the 1,264 archived data packages linked to one of the four journals and published on the Dryad website before 2014, 72 were identified as meeting the selection criteria. This sample represents a diverse range of taxa (Fig 1) and is comparable to the 73 studies surveyed by Mills et al. [2], although my methodology permits individual populations to be represented more than once, since the survey was conducted at the level of published articles (S1 Table). Of these 72 data packages, five had long-term embargoes remaining active (three packages with 5-year embargoes [15–17]; two packages with 10-year embargoes [18,19]). For two of these [17,19], the time span of the study could not be estimated because this information is not provided in the associated articles [20,21]. For a third package [22], the archived data indicated 10 years were represented (dummy coding was used to disguise factor level identities, including for year), yet the text of the associated paper suggests data collection covered a considerably greater time span [23]. However, since the study period is not stated in the text, I followed the archived data [22] in assuming data collection spanned a 10-year period. The distribution of study time spans is shown in Fig 2.

**Fig 1:**
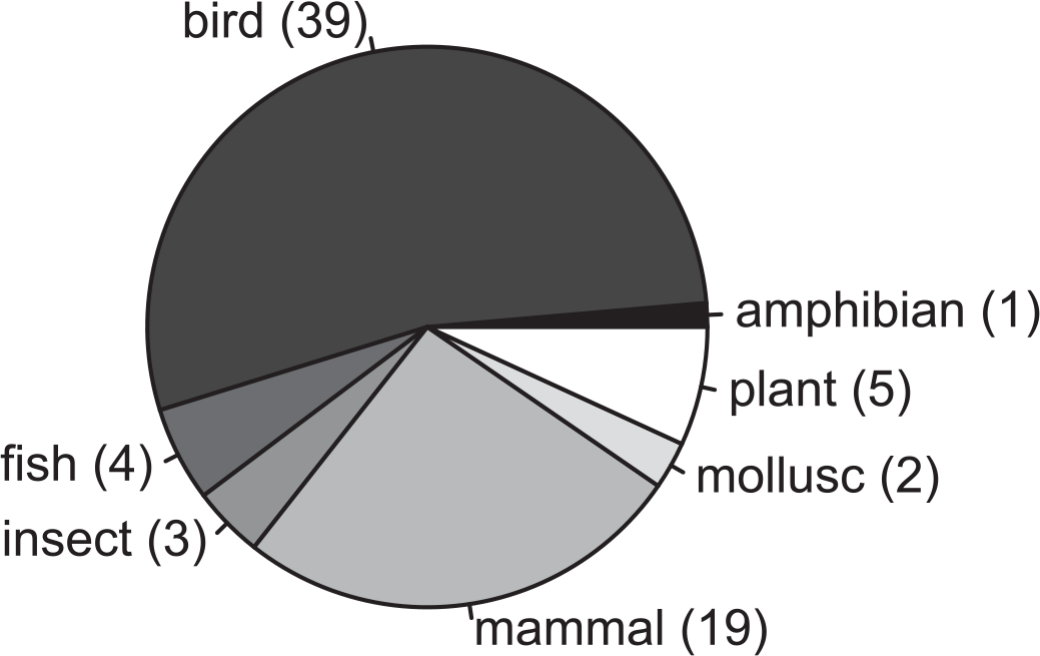
Taxonomic representation of the 72 data packages included in the survey. The number of packages for each taxon is given in parentheses (note: one data package included data describing both insects and plants [9], while other data packages represented multiple species within a single taxonomic category).

**Fig 2:**
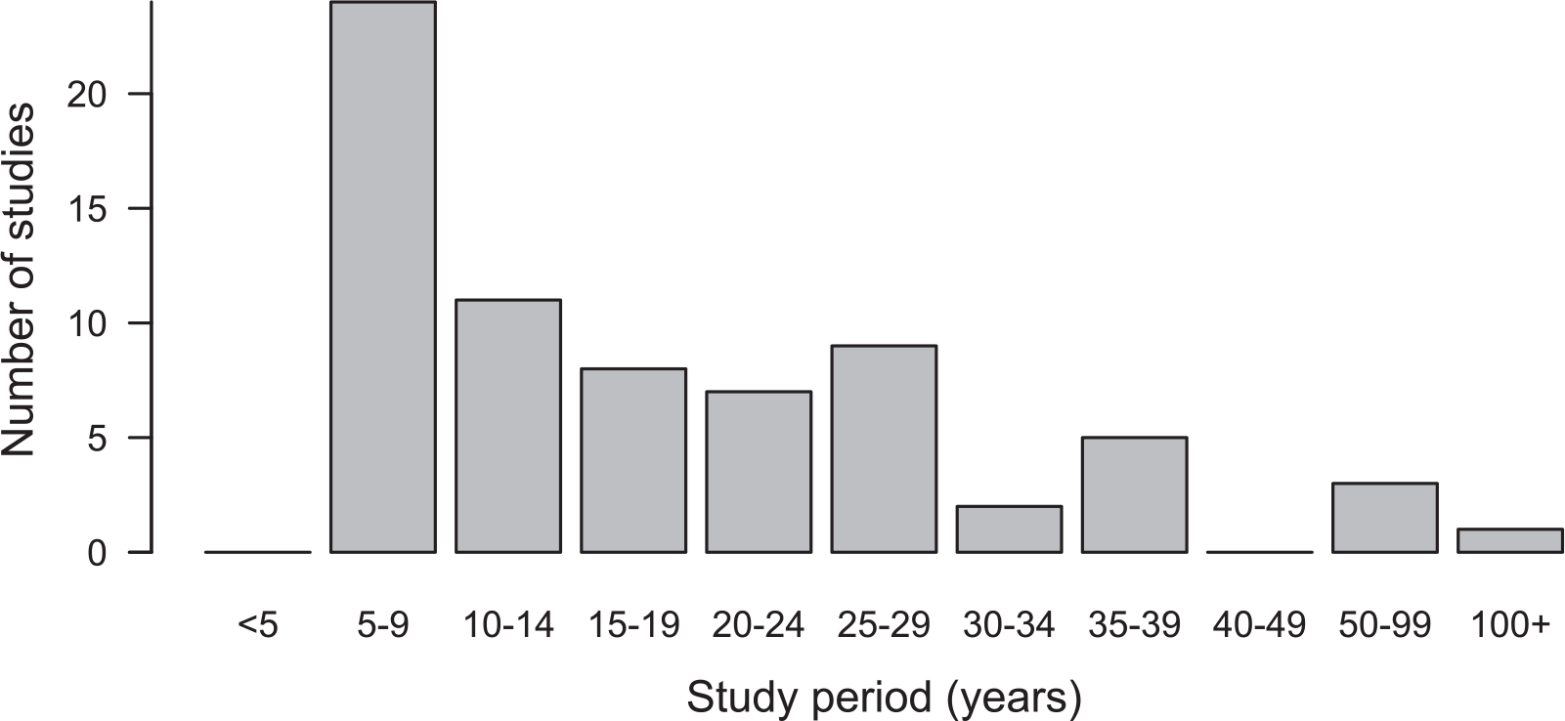
The study periods of the 70 data packages included in the survey for which this could be calculated.

For each year from 2000 to 2004, these four journals contributed no more than a single data package to Dryad between them. However, around the time that the Joint Data Archiving Policy (JDAP; [24]) was adopted by three of these, we see a surge in PDA by ecologists and evolutionary biologists (Fig 3), such that in 2015 these four journals were collectively represented by 709 data packages. Of course, Mills et al. [2] argue against mandatory archiving of primary data for long-term studies in particular. For this subset of articles published in these four journals, the same pattern is observed: prior to adoption of the JDAP, only two data packages associated with long-term studies had been archived in Dryad, but following the implementation of the JDAP as a condition of publication in *The American Naturalist*, *Evolution*, and *Journal of Evolutionary Biology*, there is a rapid increase in the number of data packages being archived, despite the continuing availability of alternative venues should authors wish to avoid the purported costs of PDA as Mills et al. [2] contend. As the editorial policy of *Proceedings B* has shifted towards an increasingly strong emphasis on PDA (it is now mandatory), there has similarly been an increase in the representation of articles from this journal in Dryad, both overall (Fig 3) and for long-term studies in particular (Fig 4). These observations suggest that authors rarely chose to publicly archive their data prior to the adoption of PDA policies by journals and that uptake of PDA spread rapidly once it became a prerequisite for publication. In this respect, researchers using long-term population studies are no different to those in other scientific fields, despite the assertion by Mills et al. [2] that they are a special case owing to the complexity of their data. In reality, researchers in many other scientific disciplines also seek to identify relationships within complex systems. Within neuroscience, for example, near-identical objections to PDA were raised at the turn of the century [25], while archiving of genetic and protein sequences by molecular biologists has yielded huge advances but was similarly resisted until revised journal policies stimulated a change in culture [1,26].

**Fig 3:**
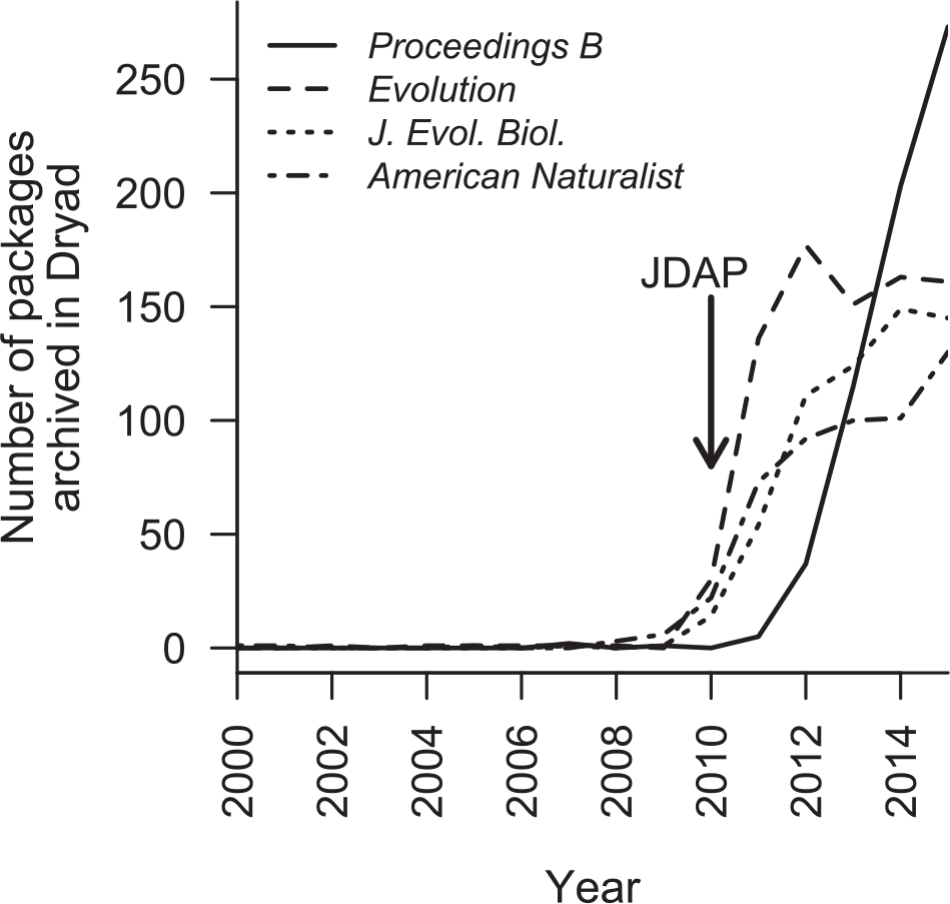
Total number of data packages archived in the Dryad Digital Repository each year for four leading journals within ecology and evolutionary biology. Arrow indicates when the Joint Data Archiving Policy (JDAP) was adopted by *Evolution*, *Journal of Evolutionary Biology*, and *The American Naturalist*. Note that because data packages are assigned a publication date by Dryad prior to journal publication (even if an embargo is imposed), some data packages will have been published in the year preceding the journal publication of their associated article.

**Fig 4:**
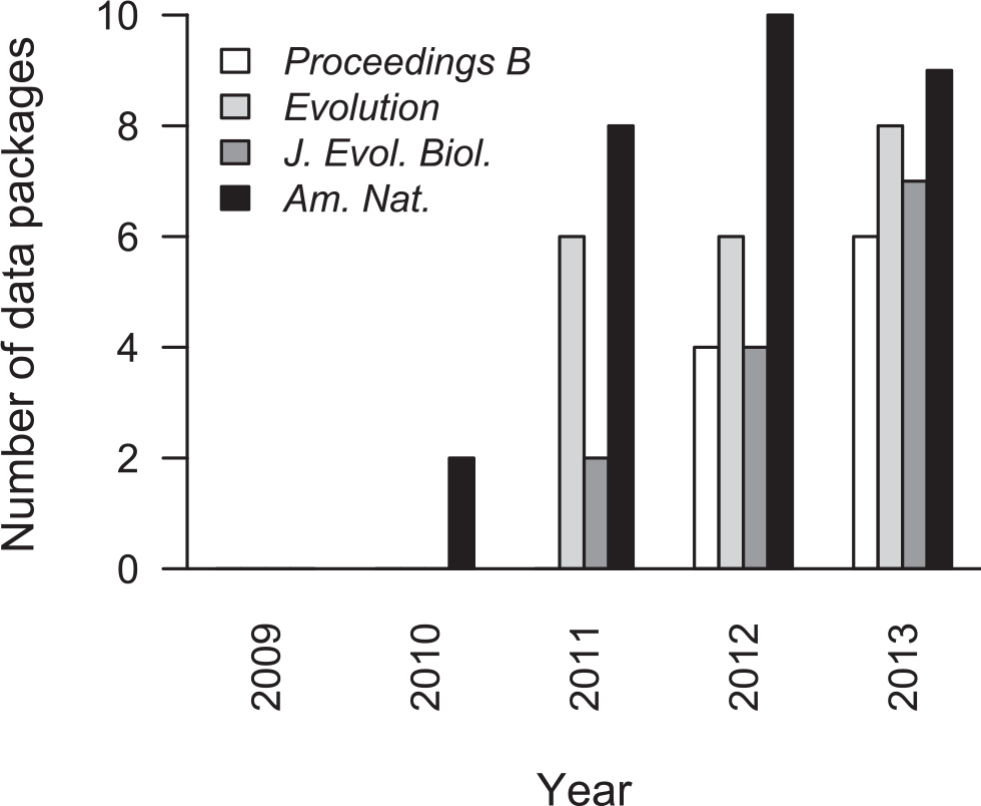
Publication dates of the 72 data packages from long-term study populations that were included in the survey.

A primary concern raised by opponents of PDA is that sharing their data will see them “scooped” by independent researchers [6,8,27–30]. To quantify this risk for researchers maintaining long-term population studies, I used the Web of Science (wok.mimas.ac.uk) to search for citations of each data package (as of November 2015). For the 67 Dryad packages that were publicly accessible, none were cited by any article other than that from which it was derived. However, archived data could conceivably have been reused without the data package being cited, so I examined all journal articles that cited the study report associated with each data package (median citation count: 9; range: 0–58). Although derived metrics from the main articles were occasionally included in quantitative reviews [31,32] or formal meta-analyses [33], I again found no examples of the archived data being reused by independent researchers. As a third approach, I emailed the corresponding author(s) listed for each article, to ask if they were themselves aware of any examples. The replies I received (*n* = 35) confirmed that there were no known cases of long-term population data being independently reused in published articles. The apparent concern of some senior researchers that PDA will see them "collect data for 30 years just to be scooped" [30] thus lacks empirical support. It should also be noted that providing primary data upon request precedes PDA as a condition of acceptance for most major scientific journals [8]. PDA merely serves to ensure that authors meet this established commitment, a step made necessary by the failure rate that is otherwise observed, even after the recent revolution in communications technology [34–36]. As my survey shows, in practice the risk of being scooped is a monster under the bed: empirical assessment fails to justify the level of concern expressed. While long-term population studies are unquestionably a highly valuable resource for ecologists [2,37–39] and will likely continue to face funding challenges [37–39], there is no empirical support for the contention of Mills et al. [2] that PDA threatens their viability, although this situation may deserve reassessment in the future if the adoption of PDA increases within ecology and evolutionary biology. Nonetheless, in the absence of assessments over longer time frames (an inevitable result of the historical reluctance to adopt PDA), my survey results raise doubts over the validity of arguments favouring extended embargoes for archived data [29,40], and particularly the suggestion that multidecadal embargoes should be facilitated for long-term studies [2,41].

Authors frequently assert that unique aspects of their long-term study render it especially well suited to addressing particular issues. Such claims contradict the suggestion that studies will become redundant if PDA becomes the norm [2] while simultaneously highlighting the necessity of making primary data available for meaningful evaluation of results. For research articles relying on data collected over several decades, independent replication is clearly impractical, such that reproducibility (the ability for a third party to replicate the results exactly [42]) is rendered all the more crucial. Besides permitting independent validation of the original results, PDA allows assessment of the hypotheses using alternative analytical methods (large datasets facilitate multiple analytical routes to test a single biological hypothesis, which likely contributes to poor reproducibility [43]) and reassessment if flaws in the original methodology later emerge [44]. Although I was not attempting to use archived data to replicate published results, and thus did not assess the contents of each package in detail, at least six packages [10,45–49] failed to provide the primary data underlying their associated articles, including a quantitative genetic study [50] for which only pedigree information was archived [47]. This limits exploration of alternative statistical approaches to the focal biological hypothesis and impedes future applications of the data that may be unforeseeable by the original investigators (a classic example being Bumpus' [51] dataset describing house sparrow survival [52]), but it seems to be a reality of PDA within ecology and evolution at present [53].

The "solutions" proffered by Mills et al. [2] are, in reality, alternatives to PDA that would serve to maintain the status quo with respect to data accessibility for published studies (i.e., subject to consent from the PI). This is a situation that is widely recognised to be failing with respect to the availability of studies' primary data [34–36,54]. Indeed, for 19% (13 of 67 nonembargoed studies) of the articles represented in my survey, the correspondence email addresses were no longer active, highlighting how rapidly access to long-term primary data can be passively lost. It is unsurprising, then, that 95% of scientists in evolution and ecology are reportedly in favour of PDA [1]. Yet, having highlighted the value and irreplaceability of data describing long-term population studies, Mills et al. [2] reject PDA in favour of allowing PIs to maintain postpublication control of primary data, going so far as to discuss the possibility of data being copyrighted. Such an attitude risks inviting public ire, since asserting private ownership ignores the public funding that likely enabled data collection, and is at odds with a Royal Society report urging scientists to "shift away from a research culture where data is viewed as a private preserve" [55]. I contend that primary data would better be considered as an intrinsic component of a published article, alongside the report appearing in the pages of a journal that presents the data's interpretation. In this way, an article would move closer to being a self-contained product of research that is fully accessible and assessable. For issues that can only be addressed using data covering an extended time span [2,37–39], excusing long-term studies from the expectation of publishing primary data would potentially render the PIs as unaccountable gatekeepers of scientific consensus. PDA encourages an alternative to this and facilitates a change in the treatment of published studies, from the system of preservation (in which a study's contribution is fixed) that has been the historical convention, towards a conservation approach (in which support for hypotheses can be reassessed and updated) [56]. Given the fundamentally dynamic nature of science, harnessing the storage potential enabled by the Information Age to ensure a study's contribution can be further developed or refined in the future seems logical and would benefit both the individual authors (through enhanced citations and reputation) and the wider scientific community.

The comparison Mills et al. [2] draw between PIs and pharmaceutical companies in terms of how their data are treated is inappropriate: whereas the latter bear the financial cost of developing a drug, a field study's costs are typically covered by the public purse, such that the personal risks of a failed project are largely limited to opportunity costs. It is inconsistent to highlight funding challenges [2,37] while simultaneously acting to inhibit maximum value for money being derived from funded studies. Several of the studies represented in the survey by Mills et al. [2] comfortably exceed a 50-year time span, highlighting the possibility that current PIs are inheritors rather than initiators of long-term studies. In such a situation, arguments favouring the rights of the PI to maintain control of postpublication access to primary data are weakened still further, given that the data may be the result of someone else's efforts. Indeed, given the undoubted value of long-term studies for ecological and evolutionary research [2,37,39], many of Mills et al.'s [2] survey respondents will presumably hope to see these studies continue after their own retirement. Rather than owners of datasets, then, perhaps PIs of long-term studies might better be considered as custodians, such that—to adapt the slogan of a Swiss watchmaker—“you never really own a long-term population study; you merely look after it for the next generation.”

## Supporting Information

S1 Table Details of the 72 data packages (and their associated articles) included in the survey. (TXT)

S2 Table The number of data packages archived in the Dryad Digital Repository each year from 2000 to 2015 for four leading journals within ecology and evolutionary biology (*The American Naturalist*, *Proceedings of the Royal Society B*: *Biological Sciences*, *Evolution*, and *Journal of Evolutionary Biology*). (TXT)

## Abbreviations

JDAP: Joint Data Archiving Policy
PDA: public data archiving
PI: principal investigator
